# Malarial Irritation of the Female Bladder

**Published:** 1886-06

**Authors:** Henry K. Leake

**Affiliations:** Dallas, Texas


### A CASE OF MALARIAL IRRITATION OF THE BLADDER IN THE FEMALE.

*By Henry K. Leake, M. D. Dallas, Texas.*

[Abstract of a paper read before the Texas State Medical Association. F. E. D.]

I desire to record an observation, which I have recently made, exemplifying the effect that the malarial poison may exert upon the female bladder; an observation which may appear common place, since, as is well known, it has not escaped mention by Prof. Skene in his excellent work on the diseases of the bladder and urethra in the female, as well as by other authors of equal or less prominence, who have attended to the same subject.

Nevertheless, considering the mere allusions by these writers to irritation of the bladder in women, which may be caused by the presence of malaria in the system, on account doubtless of the rare occurrence of this affection, it may be questioned whether the latter has been sufficiently individualized as a distinct and independent malady, deserving especial prominence in the nosology of diseases of the bladder, which seriously disturb the functions of this sensitive viscus. There is the additional reason also for reporting the experience which I have had of this peculiar and interesting disorder, in the fact, that much obscurity yet surrounds the entire subject of disturbance of the functions of this organ in the female, the integrity of which is so vital to the comfort, happiness and safety of the individual.

Moreover, such conditions often tax the diagnostic acumen of the physician to the utmost, and even when by the exclusive method, rigorously employed, many causes of irritation of the bladder may be eliminated from the problem in hand, there will yet remain in particular cases, other causes which may elude discovery, thus obscuring the pathogeny and defeating every measure of treatment, which is attempted.

About March 1st of the present year, a lady whose health has been uninterruptedly good, thirty-seven years of age, the mother of six children, the last of which being an infant of four months, applied to me for treatment, for what, she considered the ailment to be, incontinence of urine. She stated that the condition had come on gradually, at the first amounting to a mere frequency of urination during the day, without any attendant pain or other symptom, which attracted her attention. This frequency had increased, however, to such an extent as to seriously embarrass her in the performance of domestic duties, and prevent her from visiting friends, or doing neeessary shopping. Moreover, she soon became troubled at night, often rising six or perhaps a dozen times in obedience to the urgent calls for micturition.

The amount of urine passed at each discharge was not large, bnt exceeded in quantity that ordinarily retained in cases of acute cystitis, which the affection in many respects closely resembled.

There were no deposits in the urine worth noting. It appeared to be somewhat higher colored than normal. There was also a super-abundance of mucus, in the form of large floculi, but no pus or blood.

As the case progressed, the desire to evacuate the bladder was preceded by a sharp twinge of pain, which the patient averred was "low down at the very neck of the bladder," but which was immediately relieved on emptying the viscus. There was no tenderness at any point except a slight pain, experienced when the neck of the bladder was firmly pressed towards the pelvis.

The frequency of micturition increased to almost constant dribbling from the bladder, both daily and nocturnal; the cloud of mucus in the urine was much augmented, and while the color appeared to remain unchanged, there was evidently a large excretion of solid matter composed probably of phosphates.

The uneasiness elicited at the neck of the bladder by pressure on this part soon changed to actual soreness. At the end of the second week the case had passed into one of apparently serious import, and was operating with telling effect on the vitality and mental equipoise of the patient.

The tripod of treatment, namely, rest, opium and alkalies, upon which Van Bnren and Keys cogently protest the successful management of cystitis rests, was relied on to relieve what I now feared was a case of this distressing disease, the cause of which I could not then determine. The constitutional effect of belladonna was evoked also to mitigate the symptoms, and finally hot water vaginal injections were employed for their well known analgesic and antiphlogistic effects upon the pelvic viscera.

Such measures gave only temporary relief, the features of the case resuming their original character whenever the effect of medication, which was occasionally suspended to ascertain the *status quo* of the disease, had passed off.

At the beginning of the third week from the first appearance of the symptoms, the patient complained of slight chilliness towards evening, and it was observed that this was followed by fever, the thermometer in the mouth registering 101 degrees. These symptoms were interpreted to indicate the constitutional expression of the local inflammation existing in the bladder. Hence no special attention was directed towards them.

The chilliness was repeated however on the third evening.

and on the fourth day at the same hour reappeared as the prodrome of a marked rigor, followed by an abrupt rise of temperature of 103 degrees succeeded by sweating and a return to the normal temperature in about four hours, thus clearly demonstrating a well defined periodicity of the febrile movement.

Suspicion being now aroused as to the essential nature of the case, the patient was promptly placed on ten grain doses of the sulphate of quinine to be taken every four hours with mercurial and saline purgatives, the latter being indicated by the appearance of the tongue, and the confined state of the bowels, which was due not altogether to the opium administered, since this physical modifier had been exhibited both freely and simultaneously.

The substitution of the quinine for the treatment previously pursued, like the fabled wand of the magician, broke the spell of enchantment, which, by its subtle and potent influence had held the patient with relentless grasp for three weeks, and had transformed a hopeful and contented disposition into one of melancholy and apprehension.

At the end of four days from the administration of the first dose of quinine the patient was virtually convalescent. During this period no opiate was employed, nor any other medicine but quinine taken, save an occasional dose of neutral mixture, chiefly for its sudorific effect. Nevertheless, the irritation of the bladder did not return, and the close of the week found the patient, although debilitated by the trying ordeal through which she had passed, enabled to resume her accustomed duties.

[We regret that we cannot insert the Doctors' interesting comments on the case for want of room, Ed.]

